# Hepatoprotection of *Cinnamomum burmannii* ethanolic extract against high-fat and cholesterol diet in Sprague–Dawley rats (*Rattus norvegicus*)

**DOI:** 10.14202/vetworld.2022.930-936

**Published:** 2022-04-15

**Authors:** Retno Susilowati, Abdul Malik Setiawan, Afida Fatimatuz Zahroh, Zadani Nabila Ashari, Alifatul Iffiyana, Ricky Hertanto, Muhammad Basyarudin, Isnaeni Hartiningsih, Mahrus Ismail

**Affiliations:** 1 Department of Biology, Faculty of Science and Technology, State Islamic University of Maulana Malik Ibrahim Malang, Malang 65144, East Java, Indonesia; 2 Department of Microbiology, Faculty of Medicine and Health Sciences, State Islamic University of Maulana Malik Ibrahim Malang, Malang 65144, East Java, Indonesia; 3 Department of Chemistry, Faculty of Science and Technology, State Islamic University of Maulana Malik Ibrahim Malang, Malang 65144, East Java, Indonesia

**Keywords:** *Cinnamomum burmannii*, hepatoprotection, malondialdehyde, steatohepatitis, superoxide dismutase

## Abstract

**Background and Aim:**

The pathogenesis of non-alcoholic steatohepatitis involves non-alcoholic fatty liver, oxidative stress, inflammation, and fibrosis. Although the long-term use of cinnamon bark in larger doses can negatively affect good health, proper use of its extracts effectively and efficiently improves health. Therefore, this study aimed to determine the minimal dose of *Cinnamomum Burmannii* extract through its activity in inhibiting oxidative stress in rats’ livers treated with a high-fat and cholesterol diet (HFCD).

**Materials and Methods:**

Forty-two Sprague–Dawley rats (*Rattus norvegicus*), weighing 200-250 g body weight (BW), were divided into seven treatment groups with six replications: Normal, HFCD, atorvastatin, quercetin, and *C. burmannii* ethanol extract group, after which they were administered different dosages (i.e., 100, 200, and 300 mg/kg BW). Except for the normal group, rats were concomitantly administered HFCD with each treatment for 21 days. Then, their malondialdehyde (MDA) levels and superoxide dismutase (SOD) activity were assessed using colorimetry. However, their steatosis levels were determined based on histological preparations with hematoxylin-eosin staining.

**Results:**

Duncan’s multiple range test (DMRT) results indicated that all treatments had a significantly lower MDA than HFCD and normal rats (a=0.01). DMRT results also showed that treating with the *C. burmannii* ethanol extract at all dosages resulted in a significantly higher SOD activity level in HFCD rats than those treated with quercetin and atorvastatin (a=0.01). Furthermore, results showed that treatment with *C. burmannii* extracts at a dosage of 300 mg/kg BW incredibly maintained SOD activity as effective as quercetin, atorvastatin, and normal rats. Besides, while steatohepatitis levels of *C. burmannii* ethanol extract at dosages of 200 and 300 mg/kg BW commensurated with normal rats, steatohepatitis levels were significantly lower than those administered other concentrations or treatments (a=0.05).

**Conclusion:**

Ethanolic *C. burmannii* extracts protected the liver by regulating oxidative stress. Therefore, a 200 mg/kg BW dose is proposed as the minimal hepatoprotection dose to prevent fatty liver formation.

## Introduction

Non-alcoholic fatty liver disease (NAFLD) refers to a heterogeneous group of liver diseases characterized by excessive fat [1], especially triglyceride accumulation in an individual’s liver without alcohol consumption, viral infection, or drugs that can induce a steatotic liver [2]. NAFLD covers a broad spectrum of liver diseases ranging from a simple steatotic liver NAFL and non-alcoholic steatohepatitis (NASH) to liver fibrosis and ultimately cirrhosis and hepatocellular carcinoma [3,4]. Nevertheless, in NAFL and NASH, liver disease phases are reversible and can return to normal if adequately treated and managed [2]. Specifically, obesity and medical conditions, such as hypercholesterolemia and diabetes, are risk factors for developing NAFL and NASH [5].

Liver tissue is sensitive to the lipase hormone. The higher the lipid intake to the liver tissue, the higher lipolysis occurs, producing free fatty acids (FFAs). Therefore, an overload of FFA within liver cells increases beta-oxidation, followed by mitochondrial dysfunction and endoplasmic reticulum stress. NADPH oxidase activation is the primary cause of an increase in reactive oxygen species (ROS) production, such as O_2_, H_2_O_2_, malondialdehyde (MDA), and 4-hydroxy-2-nonenal (HNE). Moreover, beta-oxidation produces more ROS [6]. In addition, NAFLD with mitochondrial dysfunction triggers oxidative stress when ROS overproduction occurs. However, in the subchronic phase, ROS stress leads to liver inflammation. This condition is the undergirding factor accounting for fibroblast cell migration and hepatic stellate cell differentiation into myofibroblasts found in NASH livers [7]. Besides, connective tissue formation of collagenic fibrous tissue is the cause of hepatitis cirrhosis [6-9].

High ROS levels increase the oxidative reaction of various compounds in the body. Hence, increased peroxide lipids, such as MDA, TBAR, 4-HNE, and the rise of 8-ODHdG as the DNA damage product, signify oxidative stress [6]. A subsequent increase in ROS production without enhanced enzymatic oxidants, including a decreased output of antioxidant enzymes, such as superoxide dismutase (SOD), glutathione peroxidase (GPX), and catalase, also indicates oxidative stress. Thus, exogenous antioxidant intake is necessary. SOD is the enzyme that plays the most significant crucial role in superoxide radical deactivation, followed by GPX and catalase.

Traditional herbal medications have garnered improved interest in supplying therapeutic candidates to treat NAFLD [10]. These medications include goji berry, green tea, resveratrol, milk thistle, and garlic [11]. A study reported that the daily intake of gallic acid protected against hepatic steatosis, weight problems, and hypercholesterolemia. Gallic acid in garlic can also reduce insulin resistance in HFD-administered NAFL mice. In that study, gallic acid reduced impaired glucose and lipid homeostasis in a high-fat weight reduction plan triggered NAFLD mice [12]. Similarly, cinnamon bark extract is also proposed to improve health in overweight subjects by suppressing serum insulin [13]. It was reported that the oral administration of two active cinnamon extracts, that is, cinnamaldehyde and costunolide, significantly decreased plasma glucose, glycated hemoglobin, total cholesterol, low-density lipoprotein cholesterol, triglyceride, and increased levels of reduced hepatic GPX and SOD activity. Furthermore, cinnamaldehyde and costunolide restored altered plasma levels of alanine aminotransferase, aspartate aminotransferase, creatinine, and uric acid to normal [14].

Some major components of *Cinnamomum burmannii* oil are extracted from its bark, including trans-cinnamaldehyde (77.06-84.71%), cinnamyl acetate (12.65-15.59%), cinnamyl alcohol (0-4.56%), and cinnamic acid (3-8%) [15]. Previously, it was reported that the ethanol extracted component of *C. burmannii bark* comprised €-cinnamaldehyde, (Z)-cinnamaldehyde (52.48-82.62%), proanthocyanidin (12.08%), coumarin (1.97-7.31%), and cinnamyl alcohol (0.41-6.06%) [16]. Moreover, alcohol-based extracts from the bark of *C. burmannii* originated from Bogor, Indonesia, contained active flavonoid compounds, including 12.4% cinnamaldehyde, 15.1% transcinamaldehyde, and 0.003% coumarin [17]; 14.63±4.3 mg/g quercetrin, 18.76±4.8 mg/g quercetin [18]. In addition, former researchers reported that the alcohol extract of *C. burmannii* from Bogor possessed a high antioxidant activity with IC50 as high as 1.273 ppm [17]. However, water extracts of *C. burmannii* increased SOD activity, followed by decreased lipid peroxide. Nevertheless, it did not affect the catalase activity. Furthermore, as a natural product, the water extract of *C. burmannii* potentially decreased pro-inflammatory cytokine mRNAs; interleukin (IL)-6, IL-1β, cyclooxygenase-1, and tumor necrosis factor alpha [19], including pro-inflammatory cytokines IL-8, Toll-like receptor (TLR)-2, and TLR-4 [20].

Nowadays, high lipid consumption is unavoidable since various high lipid, cholesterol, and fructose products exist. These three compounds potentially cause fatty liver and its complications during long-term consumption. Nevertheless, consuming antioxidant-rich and antihyperlipidemic materials as a side dish to junk foods in the appropriate dosage can inhibit fatty liver occurrence. Similarly, although cinnamon bark can have an adverse effect in larger doses or long term [21], adequate use of the cinnamon extract can effectively and efficiently manage health.

Therefore, this study minimized the concentration of alcohol used to extract bioactive compounds from *C. burmannii* bark to evaluate its hepatoprotection abilities through effects on MDA, SOD, and liver lipidation levels in Sprague–Dawley Rats (*Rattus norvegicus*) induced by a high-fat and cholesterol diet (HFCD).

## Materials and Methods

### Ethical approval

Animal maintenance and handling followed Principles of Laboratory Animal Care (NIH publication no. 85-23, revised 1985) [22]. The ethics commission approved all the protocols for animal study No. 015/EC/KEP. FST/2018. The Biorat feed was standard chow diet and water *ab libitum*, HFCD given 20 g/day. Laboratory of Indonesia’s National Agency for Drug and Food Control provides male *R. norvegicus* Sprague–Dawley Rats as the animal model, and those are healthy and microbial contaminant free.

### Study period and location

The study was conducted from April 2020 to August 2021 at the Laboratory of Animal Physiology and Biomolecules, Faculty of Science and Technology, State Islamic University Maulana Malik Ibrahim Malang, East Java, Indonesia.

### Materials

PT Citra Ina Feedmill, Jakarta, Indonesia, provided the Biorat feed. Meanwhile, PT Saktisetia Sentosa, Indonesia, provided the butter. Kimia Pharma Indonesia also provided propylthiouracil (PTU). CV Gamma Scientific Biolab (Indonesia) provided cholesterol from Dyets Inc. (USA) and cholic acid from Tokyo Chemical Industry (Japan). Quercetin was obtained from Carbosynth Limited (UK). Atorvastatin was obtained from Dexa Medica Production (Indonesia). The *C. burmannii* bark simplicia was from a 12-20-year-old tree. Subsequently, the sample was powdered to a maximum size of 177 microns and sorted using an 80 mesh sieve obtained from Balai Materia Medica, East Java, Indonesia.

### Extract preparation

*C. burmannii* simplicial powder was soaked in 96% alcohol (solvent) using the maceration method. Then, a Buchner funnel and Whatman paper number 20 were used to obtain refined *C. burmannii* extracts. Subsequently, the filtrate was evaporated using a vacuum rotary evaporator at specific work conditions (water bath temperature; 60°C, flask temperature; 35-40°C, vacuum pressure; 175 mba, and rotation speed; 1 RFC).

### Experimental design

Sixty male rats of the Sprague–Dawley strain aged 35-45 days with body weight (BW) of 125-150 g were fed Biorat through *ab libitum* for approximately 60 days. However, while 42 rats met the experimental requirement of 200-250 g BW for this research, the other 18 rats were excluded from the study. Subsequently, the rats were grouped into seven treatments, each with six replications, comprising the normD (normal diet); HFCD without extract treatment but with dimethyl sulfoxide (DMSO) 1%); Ator (10 mg/kg BW atorvastatin) [23]; Quer (30 mg/kg BW quercetin) [24]; Cin300 (300 mg/kg BW *C. burmannii* extract) [25], Cin100 (100 mg/kg BW *C. burmannii* extract); and Cin200 (200 mg/kg BW *C. burmannii* extract) groups. In this study, all treatments were administered concomitantly with HFCD for 21 days.

Atorvastatin, quercetin, and the extract of *C. burmannii* with 1% DMSO were given through a gavage feeding tube at a suitable dosage of as much as 3 mL. These treatments were given 3 h after administering the HFCD between 10.00 and 11.00 a.m. every day for 21 days. Furthermore, fatty liver rats were induced using HFCD treatments, containing 50% Biorat, 20% butter, 7% quail yolk, 20% goat fat, 2% cholesterol, 1% cholic acid, 1% salt, and 0.01% PTU [18-20]. Then, the normal Biorat feed administered contained 12% water, 20% protein, 4% fat, 4% cellulose, 12% calcium, and 0.7% phosphor.

### Preparation of rat livers

After the 21^st^ day, treated rats were subjected to a fast for 8-10 h, after which they were anesthetized using ether solution, followed by euthanization through the neck disengagement. Then, liver tissue samples were separated and washed using Ringer’s solution. Subsequently, liver lysates were prepared immediately.

### Preparation of hepatic lysates

One hundred milligrams of the liver tissue sample were homogenized in a tube using a pistil at 2-4°C. The homogenization process was conducted using 0.01 M phosphate-buffered saline (PBS) at pH 7.4 and a ratio of 1:9 (w: v) until a homogenate was obtained. Then, the homogenate was centrifuged at 805 RFC for 10 min to obtain the liver lysate from the supernatant, which was subsequently stored at −20°C.

### Preparation of hepatic histology

A 5 mm×5 mm×10 mm dimension of liver tissue was washed in PBS and fixed in 10% formalin for 24 h. Next, the specimen was processed using the paraffin method. First, the paraffin block was sliced using a microtome 5 μm thick. Then, it was stained using hematoxylin-eosin [26].

### Measurement of hepatic MDA

MDA measurements within the liver lysate were conducted using a QuantiChrom™ TBARS assay kit (DTBA-100, BioAssay Systems, USA). Then, MDA concentrations were measured using a spectrophotometer (Shimadzu, UK) at 535 nm, following the procedure mentioned in the reagent kit.

### Measurement of hepatic SOD activity

Measurement of SOD enzyme activity within the liver lysate was conducted using an EnzyChrom™ SOD assay kit (Catalog No: ESOD-100, BioAssay Systems). Then, SOD concentrations were obtained using a spectrophotometer (Shimadzu) at 440 nm, following the procedure mentioned in the reagent kit.

### Hepatic steatosis observations

Hepatic steatosis examinations were conducted through liver histology preparations aided with ImageJ Software, using an Optilab digital microscope with 400×. Then, subsequent calculation of the cell percentage that underwent microvesicular and macrovesicular steatosis of several hepatocyte cells was conducted in five fields of view to determine the hepatic steatosis level of each treatment. Finally, characteristics of hepatic histopathology were explained, concurring with Lackner’s report [27].

### Statistical analysis

Each test information was presented as mean±standard deviation. Then, an examination of variance was conducted after data met the necessity for normal distribution and homogenous variance. Finally, the ordinariness of information conveyance, normality data, the homogeneity of variance, analysis of variance (ANOVA), and Duncan’s multiple range test (DMRT) were conducted using statistical package for the social sciences (SPSS) 16.0 for Windows (SPSS Inc., Chicago, IL, USA), and p<0.01 was set as the significance level.

## Results

**Figure-1:**
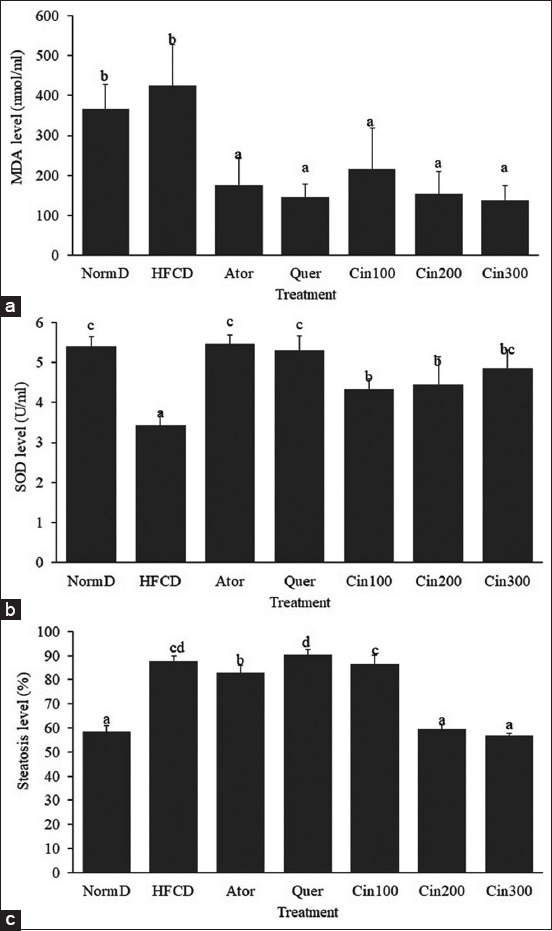
(a-c) Superoxide dismutase activity, malondialdehyde, and steatosis level of rats’ liver with HFCD. normD=Normal diets, HFCD=High-fat and cholesterol diets without treatment, Ator=Treated atorvastatin 10 mg/kg BW, Quer=Treated quercetin 30 mg/kg BW, Cin100=Treated *C. burmannii* extract 100 mg/kg BW, Cin200=Treated *C. burmannii* extract 200 mg/kg BW, Cin300=Treated *C. burmannii* extract 300 mg/kg BW. Different label means significant different in Duncan multiple rank test on α= 0.01. *C. burmannii*=*Cinnamomum burmannii*, BW=Body weight.

MDA levels of rats’ livers treated with HFCD followed the normal distribution and were homogenous (p>0.05). Moreover, one-way ANOVA showed that the treatment significantly affected liver SOD levels (p<0.01). Furthermore, DMRT results depicted that the ethanol extract of *C. burmannii* treatment on all dosages, including quercetin, and atorvastatin treatments, significantly prevented MDA formation. Besides, notwithstanding those rats were treated with HFCD, rats’ MDA levels were lower than those with a regular diet (Table-1 and Figure-1a).

**Table 1:** Effect of *C. burmanii* extract on MDA level, SOD activity, and steatosis level of rats liver with high fat and cholesterol diets.

Treatment	MDA level (nmol/mL)	SOD activity (U/mL)	Level of simple steatosis (%)
normD	366.6±62.7	5.4±0.25	58.5±2.55
HFCD	424.9±103.9	3.43±0.32	87.9±1.92
Ator	174.9±70.2	5.46±0.23	82.7±3.13
Quer	144.9±33.3	5.31±0.35	90.2±2.17
Cin100	215.7±102.9	4.33±0.27	86.5±3.76
Cin200	152.7±55.5	4.45±0.69	59.5±1.85
Cin300	138.1±35.1	4.86±0.45	56.9±0.84
Statistic test			
Normality of data	p>0.05	p>0.05	p>0.05
Homogeneity of variance	p>0.05	p>0.05	p>0.05
Analysis of Variance	P<0.01	P<0.01	P<0.01

normD=normal diets; HFCD=high fat and cholesterol diets without treatment; Ator=treated with atorvastatin 10 mg/kgbw; Quer=treated with quercetin 30 mg/kg bw; Cin100=treated with cinnamomum extract 100mg/kg bw Cin200= treated with cinnamon extract 200 mg/kg bw; Cin300=treated with cinnamon extract 300 mg/kg bw

### SOD level

Observed SOD results showed that the analyzed data, especially the HFCD, were normally distributed and were homogenous (p>0.05). However, one-way ANOVA proved that the treatment significantly affected liver SOD levels (p<0.01). Furthermore, DMRT results showed that treatments with *C. burmannii* ethanol extract at all dosages, including quercetin and atorvastatin, significantly prevented the decrease in liver SOD activity of rats with HFCD. Therefore, administering 300 mg/kg BW of *C. burmannii* extract was proven to prevent reductions in SOD activity as effective as quercetin and atorvastatin. This result was similar to the SOD activity in normal diet rats (Table-1 and Figure-1b).

### Hepatic steatosis

**Figure-2:**
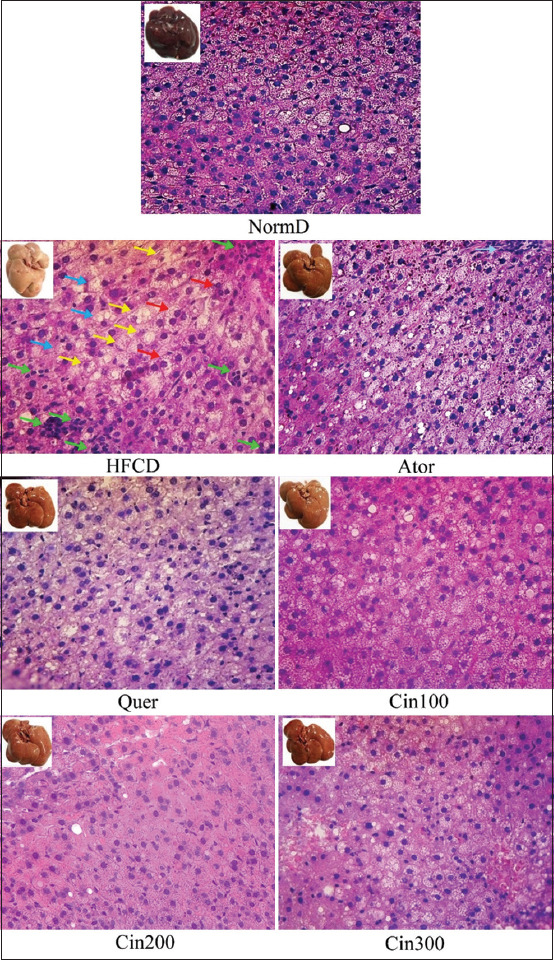
Morphologic and histological feature of rat liver steatosis with high-fat and cholesterol diets. Normal: Simple steatosis, hepatocytes with microvesicular, central nucleus, no visible inflammation. HFCD: Peripheral nuclear microvesicular steatosis (red arrow), ballooning of cells (blue arrow), inflammation with mononuclear infiltration (green arrow), and blanching of the cytoplasm some containing small Mallory-Denk (yellow arrow), Ator: Steatosis, hepatocytes with microvesicular, central nucleus, mononuclear inflammatory cells are seen, Cin100 and Cin300: Simple steatosis, hepatocytes with microvesicular, central nucleus, no visible inflammatory cells. Cin200: Normal hepatocytes. Morphologically, liver color increases with doses. The preparations were observed using hematoxylin-eosin. Staining on 400×, normD=Normal diets, HFCD=High-fat and cholesterol diets without treatment, Ator=Treated atorvastatin 10 mg/kg BW, Quer=Treated quercetin 30 mg/kg BW, Cin100=Treated *Cinnamomum* extract dose 100 mg/kg BW, Cin200=Treated *Cinnamomum* extract dose 200 mg/kg BW, Cin300=Treated *Cinnamomum* extract dose 300 mg/kg BW. BW=Body weight.

The administration of HFCD for 21 days increased hepatic steatosis levels both morphologically and histologically. As observed, the liver of rats with HFCD was pale, improving steatosis (Figure-2). However, atorvastatin, quercetin, and *Cinnamomum* extract consumption during HFCD inhibited steatosis. Based on the observation results of liver lipidation levels, data were normally distributed and homogenous (p>0.05). Moreover, one-way ANOVA results showed that the treatment significantly affected liver lipidation levels (p<0.01). Furthermore, DMRT results indicated that the administration of *C. burmannii* ethanol extracts at dosages of 200 mg/kg BW and 300 mg/kg BW significantly prevented liver lipidation. These concentrations were the same in standard diet rats. Therefore, both dosages were potent in avoiding fatty liver and even better than administering atorvastatin and quercetin (Table-1 and Figure-1c).

## Discussion

A high-fat diet increases lipid catabolism so that excess acetyl coenzyme A and FFA are released. This condition, in turn, leads to mitochondrial dysfunction and elevated ROS production. Thus, an imbalance of ROS and SOD occurs. Subsequent ROS leakage from the mitochondria results in the oxidation of these various compounds in the body. For instance, lipid results in Lipid, protein, lipoprotein, and DNA [8]. Therefore, an individual who experiences lipidemia without proper medication will face health conditions because of the development of fatty liver disease, which causes liver damage with increased oxidative stress. The results of the present study showed that the liver of Sprague–Dawley rats induced with HFCD experienced oxidative stress, which was marked by a decrease in SOD activity and a considerable increase in MDA levels. Conflictingly, the administration of *C. burmannii* bark alcohol extract exhibited protection against oxidative stress as demonstrated through the maintained levels of SOD activity similar to that of normal rats. Furthermore, the low level of MDA in all treatments compared with that of the rats administered the HFCD was confirmed.

SOD activity was appropriate based on its content in the solution. It was proportional to *C. burmannii* bark extract dose used, which was rich in flavonoids; this increased SOD synthesis through the upregulation of the transcription factor nuclear factor erythroid 2-related factor 2 (NRF-2) [28,29]. Quercetin, cinnamaldehyde, and coumarin also stimulate the upregulation of NRF-2 [30-32]. The NRF-2 protein controls the transcription of various genes, encodes enzymatic antioxidants, detoxifies, and produces anti-inflammation molecules [33]. In general, the present study elucidated that *C. burmannii* bark extracts preserve the oxidation status at an efficacy similar to that of quercetin or atorvastatin. The low MDA and high SOD levels resulting from the administration of the *Cinnamomum* extract in this study support the findings of a previous study reporting that the administration of *C. burmannii* bark alcohol extract significantly increases SOD levels and suppresses MDA formation in hyperlipidemia rats’ heart muscle [25]. Furthermore, *C. burmannii* bark extract has been reported as a potent antioxidant [17] that increases the SOD activity [19]. The antioxidant activity of *C. burmannii* bark extract in preventing lipid peroxidation has also been supported by several studies mentioning that it comprises various antioxidants from flavonoid compounds, such as cinnamaldehyde, transcinamaldehyde [17], quercetrin, and quercetin [18]. Specifically, quercetin was found to be the active compound in *C. burmannii* extract that increased GSH, SOD, and catalase in the liver of diabetic mice.

The present study revealed that administering 200 and 300 mg/kg BW of *Cinnamomum* extracts showed the same level of effectiveness in suppressing oxidative stress and preventing steatosis in HFCD administered rats. Therefore, these data indicated a minimally hepatoprotective dose of the cinnamon extract (200 mg/kg BW). This dose was lower than that reported in a previous study (300 mg/kg BW) where cinnamon was used as a preventive treatment against oxidative stress on the heart [25].

Quercetin is one of the dynamic compounds in the alcoholic concentrate of *C. burmannii* [18]. Although its single structure has been reported as a hepatoprotector in NAFLDs, this fact remains unproven due to the lack of substantial information, particularly in hepatic steatosis and hepatitis [34]. Alternatively, atorvastatin has also been considered as a lipid reduction specialist and a hepatoprotector in alleviating liver damage and steatosis [35]. However, the crude concentrate of *C. burmannii*, which has not been reported in previous studies, was used in the present study at concentrations of 300 mg/kg BW. In line with histological observations, liver morphology at a dose of 300 mg/kg BW showed an improvement in color to a normal liver color like atorvastatin. and the steatosis level was superior to that of quercetin and atorvastatin treatments in the independent test. Results also showed that the crude extract of *C. burmannii* comprised several other active ingredients, apart from quercetin [16-18], that can simultaneously provide a hepatoprotective effect in experimental animals by reducing the level of steatosis, showing considerably better reduction results compared with that of single quercetin and atorvastatin treatments. This result was presumably because the active ingredients in the crude extract of *C. burmannii* were considered to work synergistically as an antisteatosis. Nevertheless, the findings of this study establish *C. burmannii* as a long-term hepatoprotector when applied at a minimal dose of 200 mg/kg BW.

## Conclusion

*C. burmannii* bark alcohol extract exerts pharmacological effects as a hepatoprotector. The mechanism of action proposed may be that this extract increases primer antioxidants by increasing the SOD activity and suppressing ROS formation. Using *C. burmannii* at a lower concentration of 200 mg/kg BW in long-term therapy is safer than the high dose of 300 mg/kg BW. However, this study did not comprehensively disclose the effective dose range and safety parameters for using *C. burmannii* bark alcohol extract as a steatosis inhibitor, which may be investigated in future studies.

## Authors’ Contributions

RS: Contributed to conceptual design, conducted the experiment, and wrote and revised the manuscript. AMS: Performed data analysis. AFZ: Conducted histological preparation observation. AI: Measured SOD and MDA levels. ZNA and RH: Maintained and treated animal model. MB: Performed histological preparation. IH: Responsible for simplica preparation and extraction. MI: Conducted technical support in parameter measurement. All authors read and approved the final manuscript.
